# Rh‐endostatin plus camrelizumab and chemotherapy in first‐line treatment of advanced non‐small cell lung cancer: A multicenter retrospective study

**DOI:** 10.1002/cam4.5526

**Published:** 2022-12-09

**Authors:** Xingxiang Pu, QianZhi Wang, Liyu Liu, Bolin Chen, Kang Li, Yu Zhou, Zengmei Sheng, Ping Liu, Yucheng Tang, Li Xu, Jia Li, Yi Kong, Fang Xu, Yan Xu, Lin Wu

**Affiliations:** ^1^ Department of Thoracic Medical Oncology, Hunan Cancer Hospital/the Affiliated Cancer Hospital of Xiangya School of Medicine Central South University Changsha China; ^2^ Department of Oncology The Third Hospital of Changsha Changsha China; ^3^ Department of Respiratory The First Hospital of Changsha Changsha China; ^4^ Department of Oncology Hengyang Central Hospital/the affiliated Hengyang Hospital of Southern Medical University Hengyang China

**Keywords:** anti‐angiogenesis, camrelizumab, immune checkpoint inhibitors, non‐small cell lung cancer, recombinant human endostatin

## Abstract

**Background:**

Clinical evidence of immune checkpoint inhibitors combined with antiangiogenic drugs in patients with advanced non‐small cell lung cancer (NSCLC) was limited. Recombinant human endostatin (rh‐endostatin), an antiangiogenic drug, and camrelizumab, an anti‐PD‐1 antibody, have been approved for the treatment of advanced NSCLC in China. This study aimed to investigate the efficacy and safety of rh‐endostatin plus camrelizumab and chemotherapy in the treatment of advanced NSCLC.

**Methods:**

Eligible patients were enrolled and received camrelizumab (200 mg, day 1) every 3 weeks and continuous intravenous infusion of rh‐endostatin (70 mg/day, days 1–3) and cisplatin combined with pemetrexed (for adenocarcinoma) or paclitaxel (for NSCLC other than adenocarcinoma) every 3 weeks. Primary endpoint was progression‐free survival (PFS). Secondary endpoints were objective response rate (ORR), disease control rate (DCR), overall survival (OS), and safety profiles.

**Results:**

Overall, 27 patients were included, and 25 patients were eligible for efficacy evaluation. For these 25 patients, ORR was 48.15% (13/27) and DCR was 85.19% (23/27). With a median follow‐up of 10.37 months, the median PFS was 8.9 (95% CI: 4.23–13.57) months. Median OS was not reached. Overall, 96.3% of patients experienced at least one treatment‐related adverse event, and grade 3 TRAEs occurred in 9 (33.3%) patients. No unexpected AEs were observed.

**Conclusion:**

Rh‐endostatin plus camrelizumab and chemotherapy showed favorable efficacy and safety profile in patients with advanced NSCLC, representing a promising treatment regimen for these patients.

## Graphical abstract

Rh‐endostatin combined with camrelizumab plus chemotherapy treating advanced NSCLC showed ORR of 48.15% and DCR of 85.19%, with a median PFS of 8.9 months. The safety profile was comparable with former studies, representing Rh‐endostatin combined with camrelizumab and chemotherapy as a promising treatment regimen in advanced‐stage NSCLC patients.

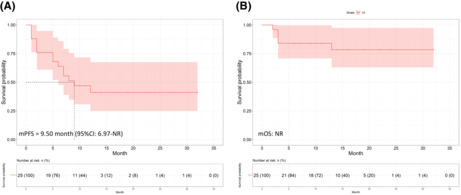

## 1 INTRODUCTION

The GLOBOCAN 2020 cancer statistics showed the incidence of lung cancer ranked second globally, while the mortality rate ranked the highest world widely, accounting for 18% of cancer‐caused death.^1^ Non‐small cell lung cancer (NSCLC) accounts for around 85% of lung cancer.^2^ Because of the high invasiveness of NSCLC and the lack of effective early screening strategies, 70% of patients with lung cancer have been diagnosed with advanced NSCLC in China.^3^ Therefore, further investigation of better treatment options remained urgent for patients.

Camrelizumab, a humanized, selective IgG4‐κ monoclonal antibody against programmed death 1 (PD‐1), has shown the efficacy and safety when combined with chemotherapy in treating NSCLC patients without driven gene alterations.^4^ Notably, immunotoxicity was also frequent in clinical practice, and more favorable combinations remained to be further unveiled. Preclinical studies indicated that immune checkpoint inhibitors (ICIs) plus anti‐angiogenesis showed synergistic antitumor effects. Anti‐angiogenesis drugs not only normalized tumor vessels but also optimized the tumor immune micro‐environment.^5^, ^6^ Similarly, results of several clinical studies showed that combining ICIs with antiangiogenic drugs in advanced NSCLC patients showed promising clinical benefits and favorable safety profile, suggesting the worth of antiangiogenic drugs and ICIs for advanced NSCLC.^7^, ^8^ These preclinical and clinical trials suggested the synergistic effect of anti‐angiogenesis agents with ICIs and achieved some results. However, optimizing the combination therapy in dosing and frequency, to optimize effectiveness, minimize toxicity and bring maximum survival benefits to patients, still needs to be further explored in clinical practice.

As a classic anti‐angiogenesis agent, recombinant human endostatin (rh‐endostatin) has shown satisfying efficacy and promising safety in patients with squamous/non‐squamous NSCLC. Rh‐endostatin has been recommended as a treatment option for locally advanced and advanced NSCLC and rh‐endostatin combined with chemotherapy for the first‐line treatment of advanced‐stage NSCLC patients without targetable genetic aberrations in Chinese NSCLC treatment guidelines.^9^, ^10^

The improvement of the delivery route of antitumor drugs could reduce the toxicities of drugs and prolong the circulation cycle of drugs in vivo. Given as a continuous intravenous infusion, rh‐endostatin could accumulate to a steady state concentration, and minimize fluctuations in drug concentrations. This could improve the efficacy of rh‐endostatin and patient's compliance.^11^, ^12^, ^13^

Currently, few studies reported the treatment for advanced NSCLC with rh‐endostatin plus camrelizumab. Considering these promising data of the potential therapeutic benefits of rh‐endostatin plus camrelizumab, we retrospectively reviewed the efficacy and safety of rh‐endostatin combined with camrelizumab in patients with advanced‐stage NSCLC in a first‐line setting. This study aimed to provide alternative treatment strategies for these patients.

## 2 MATERIALS AND METHODS

### 2.1 Study design

We retrospectively reviewed the clinical data of patients with advanced‐stage (unresectable stage IIIB and IV) NSCLC confirmed by histopathology and treated with rh‐endostatin combined with camrelizumab from December 2019 to August 2021 in four medical centers. All included patients should have received rh‐endostatin combined with camrelizumab and chemotherapy. All procedures performed in this study involving human participants have followed the ethical standards of the institutional and/or national research committee and with the 1964 Helsinki declaration and its later amendments or comparable ethical standards. This study was approved by the Hunan Cancer Hospital ethics committee (no. 202256). As this was a retrospective study, the individual informed consent was waived.

### 2.2 Patient eligibility

Male or female patients ranging from 18 to 75 years were eligible to include in this study. The inclusion criteria included: (1) patients with unresectable stage IIIB or IV pathologically confirmed NSCLC according to the classification of IASLC Thoracic Oncology, 1st Edition^14^; (2) previously untreated patients, or only received adjuvant or neoadjuvant chemotherapy for non‐metastatic tumors at least 6 months before combination treatment, or has recurrent lung adenocarcinoma 1 year after operation; (3) patients with at least one evaluable lesion according to RECIST 1.1 classification (the longest diameter on spiral CT ≥10 mm, and the longest diameter on ordinary CT ≥20 mm); (4) patients with Eastern Cooperative Oncology Group (ECOG) performance status (PS) of 0 to 2; (5) patient without EGFR mutation or ALK fusion. Exclusion criteria included: (1) pregnant or lactating women; (2) patients who had a severe acute infection and not controlled or have severe heart disease; (3) patients who had a primary brain tumor or central nerve metastasis which was not controlled.

### 2.3 Treatment plan

All included patients should have received at least two cycles of rh‐endostatin (Simcere Pharmaceutical, Jiangsu, China) combined with camrelizumab (Jiangsu Hengrui Pharmaceuticals Co, Ltd, Jiangsu, China) plus chemotherapy. According to the dosage applied by previous studies, the continuous intravenous infusion of rh‐endostatin (70 mg/day) was administrated consecutively from day 1 to day 3 and camrelizumab (200 mg fixed dose) was administrated on day 1.^15^ For patients with adenocarcinoma, the chemotherapy regimens consisted of cisplatin (DDP, 75 mg/m^2^/day) on day 1 and pemetrexed (500 mg/m^2^/day) on day 1. For patients with NSCLC other than adenocarcinoma, their chemotherapy regimens consisted of DDP (75 mg/m^2^/day) on day 1 plus paclitaxel (135 mg/m^2^/day) on day 1. All treatments were given intravenously every 3 weeks as one cycle. Symptomatic treatment or best supportive treatment was allowed according to the clinician's judgment. Computed tomography (CT) scan was conducted every two cycles to monitor the changes in lesions. Patients who experienced a tumor response or achieved stable disease (SD) after four cycles of treatment would be suggested to receive maintenance therapy of rh‐endostatin combined with camrelizumab until unacceptable adverse events (AEs) or disease progression.

### 2.4 Baseline and follow‐up assessment

Baseline assessment included gender, age, physical examination, treatment lines, and histology in this study. AEs were followed‐up and documented during treatment every week. Response evaluations were performed every two treatment cycles evaluation standard. In this study, treatment response was evaluated according to the Response Evaluation Criteria in Solid Tumors (RECIST, V1.1).^16^ Objective response rate (ORR) was defined as the percentage of patients who achieved CR and PR. Disease control rate (DCR) was defined as the percentage of patients who achieved CR, PR, and SD. Patients who received at least two cycles of combination treatment regimens with a measurable lesion were included in the response‐evaluable population. Patients who received at least two cycles of combination treatment regimens were included in the safety‐evaluable population. All patients were followed‐up throughout patient clinical data, telephone, or hospitalization record.

### 2.5 Clinical efficacy

The primary endpoint was progression‐free survival (PFS), which was defined as the interval from admission to disease progression or death. The secondary endpoint includes overall survival (OS, defined as the interval from admission to death of any cause), ORR, DCR, and AEs, which was recorded according to National Cancer Institute Common Toxicity Criteria of Adverse Events (NCI‐CTCAE) Version 3.0.^17^

### 2.6 Statistical analysis

Statistical analyses were conducted using SPSS software (version 24, IBM Software, Armonk, NY, USA) and R studio (version 4.1.2, Vienna, Austria). The visualization of the analysis results was conducted with GraphPad Prism (version 8, GraphPad Software). The categorical data were described as frequency or percentage (%), and the chi‐square test or Fisher's exact probability method was used for comparisons between groups. The median follow‐up period, PFS, and OS were analyzed by the Kaplan–Meier method. Clopper–Pearson method was used to calculate the 95% CI of ORR or DCR. *p* < 0.05 was considered statistically significant.

## 3 RESULTS

### 3.1 Baseline characteristics

**FIGURE 1:**
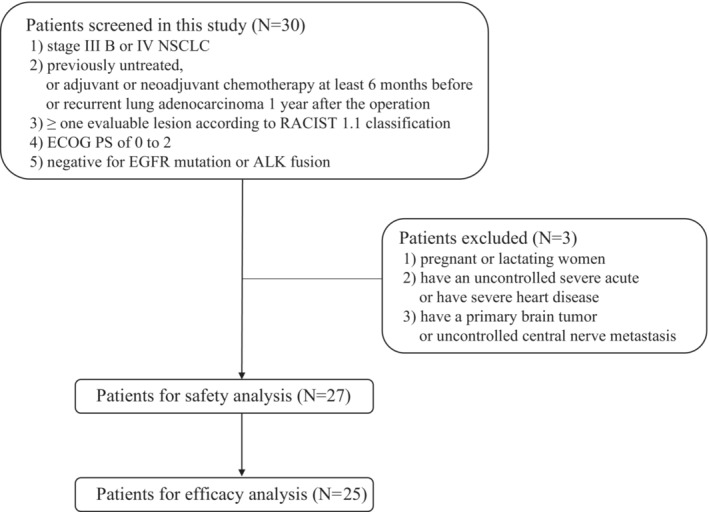
Flowchart of the inclusion and exclusion procedure.

A total of 27 eligible patients were included in safety analysis and 25 patients were included in the efficacy analysis (Figure 1). The baseline characteristics of these patients were shown in Table 1. Of the 27 patients, the median age was 57 (33–78) years. In addition, 77.8% (21/27) were male, and 22.2% (6/27) were female. For pathology type, 74.1% (20/27) were adenocarcinoma and 18.5% (5/27) were squamous. As for metastasis site, there were eight patients with pleural effusion and two patients with brain metastasis included.

**TABLE 1:** Baseline characteristics of included patients

Characteristics	Overall population (*N* = 27)
Age (year)	56.7
Gender, n (%)	
Male	21 (77.8%)
Female	6 (22.2%)
Histology, n (%)	
Adenocarcinoma	20 (74.1%)
Squamous	5 (18.5%)
Others	2 (7.4%)
Stage, n (%)	
III	2 (7.4%)
IV	25 (92.6%)
ECOG PS, n (%)	
0	8 (29.6%)
1	19 (70.4%)
Metastasis sites, n (%)	
Pleural	8 (29.6%)
Brain	3 (11.1%)
Other sites	14 (51.9%)
None	2 (7.4%)
PD‐L1 tumor proportion score, n (%)	
<1%	21 (77.8%)
≥1%	5 (18.5%)
1–49%	3 (11.1%)
≥50%	2 (7.4%)
Could not be evaluated	1 (3.7%)

Abbreviations: ECOG, Eastern Cooperative Oncology Group; PS, performance status; PD‐L1, programmed cell death ligand 1.

### 3.2 Clinical efficacy

**FIGURE 2:**
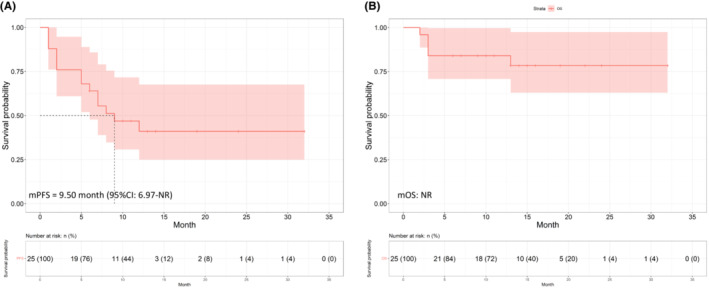
Kaplan–Meier curves for (A) progression‐free survival and (B) overall survival (OS) of the whole cohort

The data cutoff date for final analysis was September 7th, 2022, with a median follow‐up of 14.41 (95% CI: 11.52–17.30) months. Of 25 patients in response evaluation, 12 (48.0%) patients achieved PR while 10 (40.0%) patients achieved SD, resulting in an ORR of 48.0% and DCR of 88.0%. For the whole cohort, the median PFS was 7.57 months (95% CI: 5.80–NR), and the median OS was not reached (Figure 2).

**FIGURE 3:**
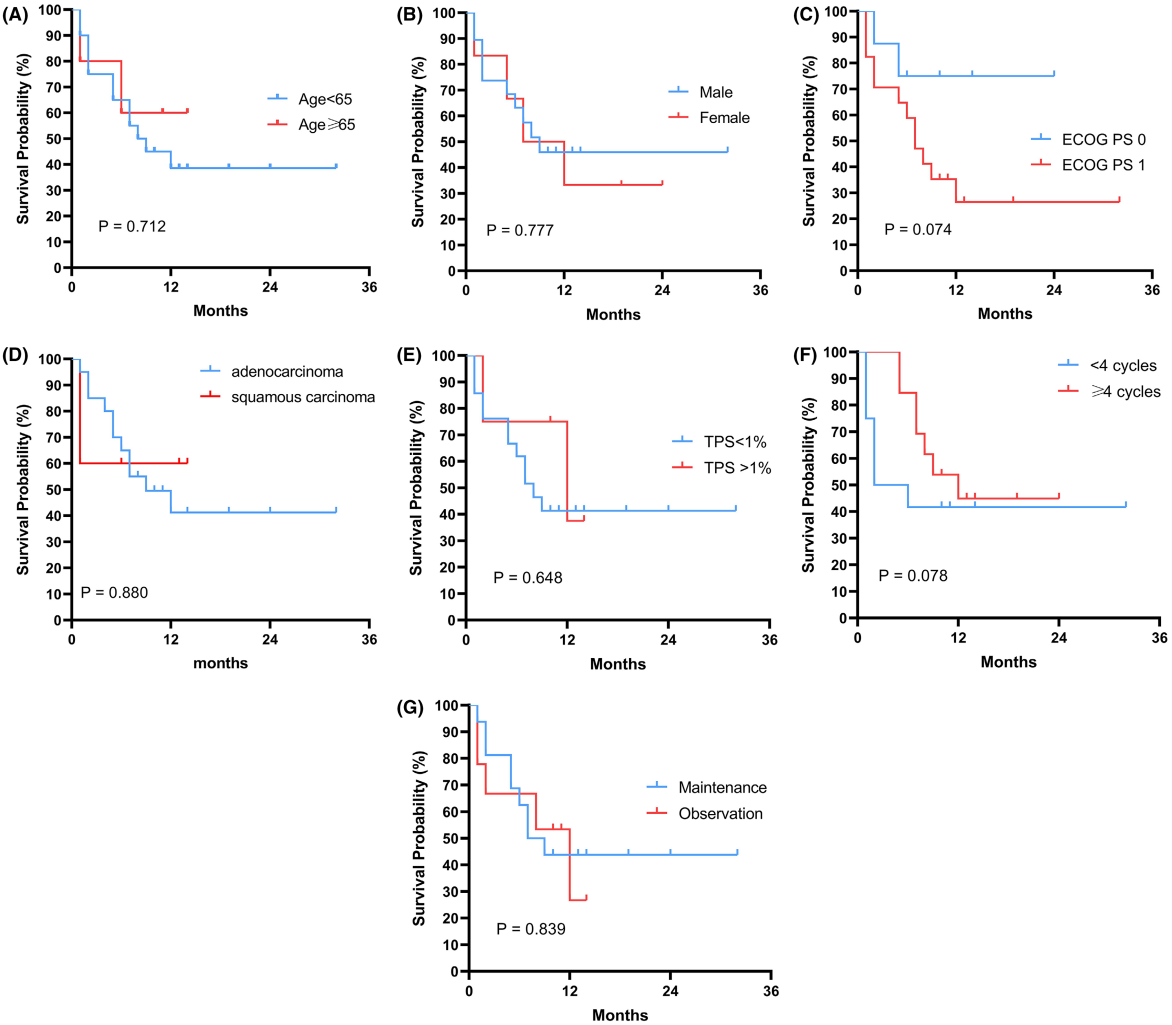
Kaplan–Meier curves for subgroup analysis of PFS. (A) Age; (B) Sex; (C) ECOG PS; (D) Pathology; (E) PD‐L1 tumor proportion score (TPS); (F) Cycles of rh‐endostatin combined with camrelizumab and chemotherapy treatment; (G) Maintenance treatment.

**FIGURE 4:**
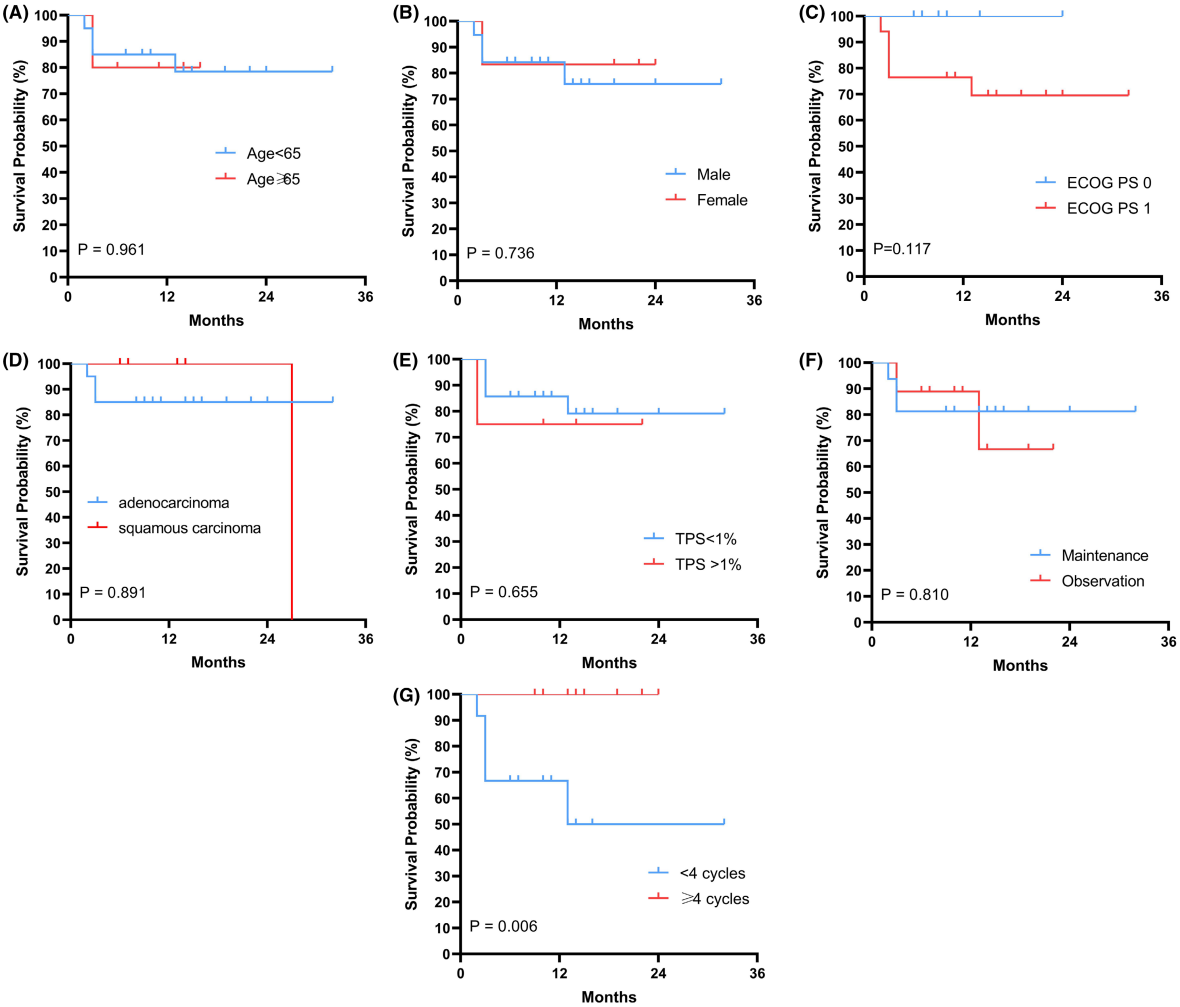
Kaplan–Meier curves for subgroup analysis of OS. (A) Age; (B)Sex; (C) ECOG PS; (D) Pathology; (E) PD‐L1 tumor proportion score (TPS); (F) Cycles of rh‐endostatin combined with camrelizumab and chemotherapy treatment; (G) Maintenance treatment.

In subgroup analyses, patients were grouped according to characteristics including age, sex, ECOG PS, treatment cycles, maintenance therapy and programmed cell death‐ligand 1 (PD‐L1) tumor proportion score (TPS). No significant differences were found between the different subgroups of age, sex, ECOG PS, or TPS in PFS and OS (Figure 3A–D,G; Figure 4A–D,G). In the subgroup of treatment cycle, of 13 patients receiving ≥4 cycles of rh‐endostatin plus camrelizumab and chemotherapy, 10 patients achieved PR, and three patients achieved SD. In the subgroup of 12 patients receiving <4 cycles of treatment, 2 patients achieved PR, and 7 patients achieved SD. Therefore, ORR was 16.7 (95% CI 2.1–48.4)% and 76.9 (95% CI 46.2–95.0)% in those patients who received <4 cycles and those who received ≥4 cycles, respectively, between which a significant difference existed (*p* = 0.007). In addition, DCR was 75.0 (95% CI 42.8–94.5) % and 100 (95% CI 75.3–100)% in those who received <4 cycles and those who received ≥4 cycles (*p* = 0.007), respectively (Table 2). Further analysis revealed that OS of those patients who received ≥4 cycles of treatment was significantly longer than that of patients who received <4 cycles of treatment (*p* = 0.06) (Figure 4F), while no significant differences was found in PFS (*p* = 0.078) (Figure 3F). For patients with pleural effusion, ORR was 50.0 (95% CI 15.7–84.3)% and DCR was 87.5 (47.3–99.7)%. For the two patients with brain metastasis, the ORR was 50.0 (95% CI 1.3–98.7)% and DCR was 100.0 (95% CI 15.8–100.0) %.

**TABLE 2:** Results of clinical efficacy

	Overall (*N* = 25), *n* (%)	<4 cycles (*n* = 12), *n* (%)	≥4 cycles (*n* = 13), *n* (%)	*p*‐value
Best response				
CR	0 (0%)	0 (0%)	0(0%)	—
PR	12 (48.0%)	2 (16.7%)	10 (76.9%)	—
SD	10 (40.0%)	6 (50.0%)	3 (23.1%)	—
PD	3 (12.0%)	4 (33.3%)	0 (0%)	—
ORR, 95% CI	48.0 (27.8–68.7)%	16.7 (2.1–48.4)%	76.9 (46.2–95.0)%	0.007
DCR, 95% CI	88.0 (68.8–97.5) %	75.0 (42.8–94.5) %	100 (75.3–100)%	0.007

Abbreviations: CR, complete remission; DCR, disease control rate; PR, partial remission; ORR, objective response rate; PD, progression disease; SD, stable disease; .

### 3.3 Adverse events

The adverse events (AEs) are outlined in Table 3. In 27 NSCLC patients included in safety analysis, 26 (96.3%) patients experienced at least one treatment‐related AE (TRAE), and grade 3 TRAEs occurred in 9 (33.3%) patients of them. The most common TRAEs were anemia, nausea, and vomiting. Especially, reactive cutaneous capillary endothelial proliferation (RCCEP), a specific AE caused by camrelizumab, occurred in 40.7% of patients, and none was ≥3‐grade AEs. No unexpected AEs were observed.

**TABLE 3:** Profile of adverse events.

Adverse events	Adverse events, (*N*, %)	
	Any grade	Grade ≥3
Hematological adverse event		
Neutropenia	8 (29.6)	2 (7.4)
Leukopenia	8 (29.6)	2 (7.4)
Thrombocytopenia	7 (25.9)	3 (11.1)
Anemia	15 (55.6)	2 (7.4)
Non‐hematological adverse events		
Cardiac creatine kinase level change	2 (7.4)	0 (0)
Nausea or vomiting	12 (44.4)	1 (3.7)
Immune‐related adverse events		
Reactive cutaneous capillary endothelial proliferation	11 (40.7)	0 (0)
Liver injury^a^	9 (33.3)	1 (3.7)
Kidney injury	3 (11.1)	0 (0)
Hypothyroidism	2 (7.4)	0 (0)
Diarrhea and colitis	0 (0)	0 (0)

^a^ One patient was considered immune‐related hepatitis.

## 4 DISCUSSION

In this retrospective multicenter study, we evaluated the efficacy and safety of the combination of rh‐endostatin with camrelizumab plus chemotherapy in patients with advanced‐stage NSCLC. Our exploratory study demonstrated that rh‐endostatin plus camrelizumab as a combined regimen resulted in a promising efficacy in improving survival outcomes and therapeutic outcomes. To the best of our knowledge, this was the first verification of the clinical outcomes and values of rh‐endostatin combined with camrelizumab plus chemotherapy in advanced‐stage NSCLC patients.

In recent years, anti‐PD‐1/PD‐L1 antibodies have shown efficacy for advanced NSCLC patients in previous clinical trials and anti‐PD‐1/PD‐L1 therapy has been recommended as a standard treatment for advanced‐stage NSCLC.^18^, ^19^, ^20^, ^21^, ^22^, ^23^ As one of the anti‐PD‐1 antibodies developed in China, camrelizumab has shown efficacy in treating advanced‐stage NSCLC and camrelizumab plus pemetrexed carboplatin was approved for the first‐line treatment of advanced non‐squamous NSCLC by National Medical Products Administration in China recently.^24^ Though anti‐PD‐1 antibody combined with chemotherapy demonstrated good efficacy in advanced‐stage NSCLC patients, some of these patients still showed unsatisfied response to this combination therapy. Also, immune‐related toxicity is frequent in clinical practice and might cause some concern of the present treatment strategies.^25^ More favorable combination therapies remained to be further explored.

Several preclinical results suggested that anti‐angiogenesis agents could enhance the antitumor effects of ICIs by both normalizing tumor vessels and improving the tumor immune microenvironment.^5^, ^26^, ^27^ Recent preclinical study verified the synergy of rh‐endostatin and anti‐PD‐1 antibody in lung cancer mouse model,^28^ which laid a solid foundation for combination therapies of rh‐endostatin plus ICIs in the treatment of advanced‐stage NSCLC. The mechanism for synergistic anti‐tumor effect of camrelizumab and rh‐endostatin varies including the activation of antigen presentation, promotion of infiltration and migration of lymphocytes and reduction of immunosuppression.^29^ Based on the above preclinical evidences, the efficacy and safety of the combination of rh‐endostatin plus camrelizumab and chemotherapy in advanced‐stage NSCLC were worth exploring.

In our study, the efficacy and survival results of rh‐endostatin with camrelizumab plus chemotherapy were satisfactory. The combination of rh‐endostatin plus camrelizumab and chemotherapy in our study achieved an ORR of 48.0% and a DCR of 88.0%. The median PFS in our study was 7.57 (95% CI: 5.80–NR) months, with median OS not reached. Previous studies have explored the efficacy and safety combination regimens of camrelizumab and other treatments, including camrelizumab and anti‐angiogenesis agents. In the CameL study, results showed that for non‐squamous NSCLC, camrelizumab plus chemotherapy achieved the median PFS for 11.3 (95% CI 9.6–15.4) months with a DCR achieving 87.8% (95% CI 82.5–92.0%).^4^ In the CameL‐Sq study, results showed that camrelizumab plus chemotherapy achieved a median PFS for 8.5 months for squamous NSCLC.^30^ For the combination of camrelizumab and anti‐angiogenesis agent, a recent study explored the efficacy of camrelizumab plus apatinib, an oral tyrosine kinase inhibitor targeting the VEGF receptor‐2, treating treatment‐naive patients with advanced‐stage non‐squamous NSCLC.^31^ In this study, the ORR was 40.0% and DCR was 92.0%, with the median PFS of 9.6 months, which was comparable to the results of our study. In addition, in the subgroup analysis of our study, for patients receiving ≥4 cycles of treatment, compared with those receiving <4 cycles of treatment, the ORR and DCR were significantly higher, with a longer OS achieved. These results preliminarily indicated that the benefits of patients may be more obvious after the combined treatment of ≥4 cycles.

Safety profile in our study was satisfying and compared to former clinical trials of camrelizumab, there were no unexpected safety events appeared in our study. Especially, RCCEP is the most symbolic adverse event related to camrelizumab. In previous multicenter prospective studies of camrelizumab, the incidence rate of RCCEP ranges from 66.8% to 79.9%.^4^, ^32^, ^33^, ^34^ In our study, the incidence rate of RCCEP was 40.7%, which was numerically lower than the above‐mentioned studies. This might contribute to the combination of camrelizumab with rh‐endostatin, as anti‐angiogenesis agent might inhibit the generation of new vessels.

There were some limitations of our study. First, the sample size of this study was small, which indicated that the results of our study should be interpreted with caution. Furthermore, enrolled NSCLC patients were with pathological heterogeneity, which might result in some uncertainty of the therapeutic effects. Therefore, larger sample sizes, multicenter, randomized, and controlled clinical trials are warranted to provide further validation for the use of rh‐endostatin and camrelizumab treating advanced NSCLC, as well as to confirm its long‐term efficacy and toxicity.

## 5 CONCLUSIONS

In summary, our study revealed that rh‐endostatin plus camrelizumab and chemotherapy has promising efficacy and safety profile in the first‐line treatment of advanced‐stage NSCLC, which indicated that this regimen could be a potential treatment option for advanced‐stage NSCLC patients. Further randomized controlled study is warranted to investigate the therapeutic outcomes of the rh‐endostatin combined with camrelizumab for advanced NSCLC.

## AUTHOR CONTRIBUTIONS

**Xingxiang Pu:** Data curation (equal); formal analysis (equal); investigation (equal); methodology (equal); validation (equal); visualization (equal); writing – original draft (equal); writing – review and editing (equal). **Qianzhi Wang:** Data curation (equal); formal analysis (equal); investigation (equal); methodology (equal); validation (equal); visualization (equal); writing – original draft (equal); writing – review and editing (equal). **Liyu Liu:** Data curation (equal). **Bolin Chen:** Data curation (equal). **Kang Li:** Data curation (equal). **Yu Zhou:** Software (equal); visualization (equal); writing – original draft (supporting). **Zengmei Sheng:** Data curation (equal). **Ping Liu:** Data curation (equal). **Yucheng Tang:** Data curation (equal). **Li Xu:** Data curation (equal). **Jia Li:** Data curation (equal). **Yi Kong:** Data curation (equal). **Fang Xu:** Data curation (equal). **Yan Xu:** Data curation (equal). **Lin Wu:** Conceptualization (equal); data curation (equal); methodology (equal); project administration (equal); resources (equal); supervision (equal); writing – review and editing (equal).

## CONFLICT OF INTEREST

The authors declare that they have no competing interests to disclose.

## Data Availability

Data included in this manuscript are available from the corresponding author under reasonable requirement.
